# Important Requirements for the Selection of Internal Standards during the Development of Desorption/Ionization Assays for Drug Quantification in Biological Matrices—A Practical Example

**DOI:** 10.3390/molecules27030690

**Published:** 2022-01-21

**Authors:** Margaux Fresnais, Seda Karabulut, Yasmin Abou Zeed, Johannes Ungermann, Julia Benzel, Kristian W. Pajtler, Stefan M. Pfister, Walter E. Haefeli, Jürgen Burhenne, Rémi Longuespée

**Affiliations:** 1 Department of Clinical Pharmacology and Pharmacoepidemiology, Heidelberg University Hospital, Im Neuenheimer Feld 410, 69120 Heidelberg, Germany; Margaux.Fresnais@med.uni-heidelberg.de (M.F.); sedaaka4155@gmail.com (S.K.); Y.Abou@stud.uni-heidelberg.de (Y.A.Z.); johannes.ungermann@stud.uni-heidelberg.de (J.U.); Walter-Emil.Haefeli@med.uni-heidelberg.de (W.E.H.); Juergen.Burhenne@med.uni-heidelberg.de (J.B.); 2 Hopp Children’s Cancer Center Heidelberg (KiTZ), Im Neuenheimer Feld 430, 69120 Heidelberg, Germany; j.benzel@kitz-heidelberg.de (J.B.); K.Pajtler@kitz-heidelberg.de (K.W.P.); s.pfister@kitz-heidelberg.de (S.M.P.); 3 German Cancer Research Center (DKFZ), Division of Pediatric Neurooncology, German Cancer Consortium (DKTK), Im Neuenheimer Feld 280, 69120 Heidelberg, Germany; 4 Department of Pediatric Hematology, Oncology and Immunology, Heidelberg University Hospital, 69120 Heidelberg, Germany

**Keywords:** desorption/ionization, mass spectrometry, drug, quantification, biological matrices

## Abstract

Desorption/ionization mass spectrometry (DI-MS) approaches allow for the rapid quantification of drugs in biological matrices using assays that can be validated according to regulatory guidelines. However, specific adaptations must be applied to create reliable quantification methods, depending on the approach and instrumentation used. In the present article, we demonstrate the importance of the molecular weight, the fragmentation pattern, and the purity of the internal standard for the development of matrix-assisted laser desorption/ionization (MALDI)-ion mobility (IM)-tandem MS and MS/MS methods. We present preliminary results of method development for the quantification of selinexor in microdialysis fluids with a stable isotopically labeled internal standard. In addition, we discuss the selection of internal standards for MALDI-MS assays using different instrumentations.

## 1. Introduction

Desorption/ionization (DI) methods offer a range of outstanding advantages for drug quantification in biological matrices [1,2], using assays which can be validated according to regulatory guidelines [1]. These advantages include the rapidity of sample preparation and analysis from any biological matrix, including fluids [3,4,5,6,7,8], and the ability to quantify drugs in their histological context when tissue sections are used [1,9,10]. Our group has previously shown that the surface properties of the biological matrix in desorption electrospray ionization (DESI) [11] or the matrix crystallization effects in matrix-assisted laser desorption/ionization (MALDI) [2,12] can strongly affect signal stability compared to electrospray ionization (ESI). For this reason, reliable normalization using appropriate internal standards (IS) is a crucial step for the successful development of fully validated methods according to regulatory guidelines [2]. Different instrumentations for DI analyses offer different options for analytical specificity and sensitivity [2]. These also allow varying degrees of flexibility in the choice of IS for normalization. Analytical strategies and mass spectrometers allowing the use of multiple reaction monitoring (MRM) and pseudo-MRM modes (using triple quadrupole (TQ) and quadrupole–time-of-flight (Q-TOF) mass spectrometers, respectively) are best suited for measuring compound-specific mass transitions. Quadrupoles permitting ion selection in a 1-Da mass window allow for (i) the precise selection of the parent ions, (ii) parent ion fragmentation and (iii) the precise selection and detection of daughter ions for downstream quantification. Cycles of measurements targeting transitions specific to the compounds of interest followed by transitions specific to the IS are performed throughout the analysis. This implies that stable signals for the targeted compounds and their IS are required throughout the analysis to avoid quantification bias due to over- or under-normalization. While this requirement is easy to fulfill with ESI, high signal reproducibility can be difficult to achieve in DI analyses [2]. Therefore, the use of DI instrumentations and approaches for the simultaneous detection of a targeted compound and an IS parent or daughter ion can be advantageous to perform a fully reliable normalization. MALDI tandem mass spectrometry (MS/MS) methods are well suited for the development of robust drug quantification assays, when instrumentations are equipped with a quadrupole permitting the simultaneous selection of the targeted compound and the IS for further fragmentation [2,9,12]. Additionally, we have proven that compared to methods based on the quantification of parent compounds, MS/MS methods were more selective and sensitive, because the background signals of the endogenous compounds were diluted [2,9,12]. However, these MS/MS strategies necessitate special consideration for the choice of IS. The fragmentation pattern of the IS is critical and signal overlap between the targeted compound and its IS should be avoided [9]. Herein, we present an example of IS candidate selection for the quantification of selinexor (SLX), a selective inhibitor of nuclear export used in cancer therapy [13], in microdialysis fluids. The importance of the molecular weight (MW), the fragmentation pattern, and the purity of the IS for MALDI-ion mobility (IM)-MS and MS/MS method development was highlighted.

## 2. Materials and Methods

### 2.1. Chemicals

**Figure 1:**
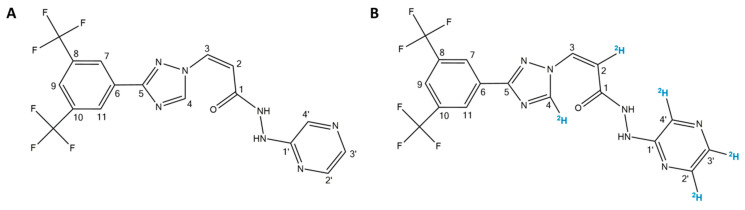
Chemical structures of (**A**) selinexor (SLX) and (**B**) [^2^H_5_]-SLX.

MS-grade water, organic solvents, and formic acid (FA) were purchased from Biosolve Chimie SARL (Dieuze, France), and 2,5-dihydroxybenzoic acid (2,5-DHB), alpha-cyano-4-hydroxycinnamic acid (CHCA), trifluoroacetic acid (TFA), bovine serum albumin (BSA), and red phosphorus were obtained from Sigma-Aldrich (Darmstadt, Germany). SLX (Figure 1A) and [^2^H_5_]-SLX (Figure 1B) were provided by Karyopharm Therapeutics Inc. (Newton, MA, USA). The certificate of analysis of [^2^H_5_]-SLX reported a chemical purity of >97% from liquid chromatography (LC)-MS analyses. LC-MS analyses also reported the incomplete incorporation of ^2^H at C2 (10% of H based on MS ratio). ^1^H-NMR analyses reported the incomplete incorporation of ^2^H at C2 (8% of H based on integration) and 12% of ^2^H incorporated at C3.

### 2.2. Choice of the MALDI Matrix for Analysis

Initial MS analyses were performed using either 50 mg/mL 2,5-DHB dissolved in MeOH/H_2_O/TFA 50:50:0.1 (*v*/*v*/*v*) or 25 mg/mL CHCA dissolved in ACN/H_2_O/TFA 70:30:0.1 (*v*/*v*/*v*). Both MALDI matrices allowed for the ionization of the two compounds of interest. However, we aimed to use the MALDI matrix that appeared the most universal for MALDI-MS assays for drug quantification. Since crystallization homogeneity is a critical parameter and CHCA displays the most homogeneous crystallization in a large range of MALDI matrices using any sample type [2], the latter MALDI matrix was used for further tests.

### 2.3. Sample Preparation

The method development aimed for the quantification of SLX in microdialysis fluid. The blank artificial biological matrix consisted of blank Ringer solution spiked with 1% BSA. Stock solutions of SLX for the different calibration (CAL) levels were prepared with serial dilution in MeOH/H_2_O 50:50 (*v*/*v*), giving concentrations from 2000 to 10 ng/mL (Table 1), and a stock solution of the IS was prepared in MeOH/H_2_O 50:50 (*v*/*v*) at 500 ng/mL. Ten microliters of the blank biological matrix was mixed with 5 µL of the reference standard (SLX) solution at the desired concentration and 5 µL of the IS stock solution. This resulted in concentrations in the biological matrix from 1000 to 5 ng/mL for the CAL samples. The sample preparation method consisted of a two-step liquid–liquid extraction (LLE) approach that was adapted for the extraction of SLX from a low volume of blank artificial biological matrix. First, 20 µL of tert-butyl methyl ether (TBME) was added to volumes of the spiked biological matrix and the batches of samples were vigorously shaken for 10 s. The samples were centrifuged for 1 min at 13,200× *g* to allow complete phase separation. One microliter from the upper layer of the LLE extract was deposited on the MALDI metal target, followed by the deposition of 1 µL of the CHCA solution.

### 2.4. Mass Spectrometric (MS) Analyses

The analyses were performed using a Synapt G2-Si instrument (Waters, Milford, MA, USA) consisting of an orthogonal-acceleration (oa) quadrupole (Q)-ion-mobility (IM)–time-of-flight (TOF) mass spectrometer equipped with a MALDI source and controlled using MassLynx v4.1 (Waters) as described in detail previously [9]. The instrument was used in resolution mode (“W” mode) and IM. IM gives peaks of higher resolution and intensity [9]. Furthermore, we previously proved that only the integration of IM data permitted the validation of drug quantification assays thanks to LC-MS-like data integration [2]. We previously described the IM-MS parameters for the analysis of parent compounds (selection of a specific parent ion in Q followed by IM separation before MS detection, referred to as method 3 in [9]) and the IM-MS/MS parameters for the analysis of fragments (selection of a specific parent ion in Q followed by collision-induced fragmentation at 32 V and subsequent IM separation of the fragments before MS detection, referred to as method 4 in [9,12]). Details for the parameters are displayed in Table 2.

The quadrupole low-mass (LM) resolution was set to 4.4 arbitrary units (a.u.), permitting ion selection in a large mass window without losing sensitivity [9]. The standard voltage value for fragmentation (32 V) was verified to be optimal for SLX, i.e., to produce intense MS/MS fragment peaks and low intensity peaks of remaining parent ions.

### 2.5. Data Processing

Mobilograms and MS spectra were extracted from MassLynx v4.1 and calibration curves were computed in Prism software version 5.01 (GraphPad, La Jolla, CA, USA). Recommendations for reporting results of IM-MS measurements [14] were followed. As IM was used here as a separation method and not for structural analyses, the drift times (DT) are reported as IM data. Two-dimensional mobility maps (mass-over-charge (*m/z*) vs. DT maps) were obtained using Driftscope version 2.9 (Waters). The previously described MobA method [9] was used for data extraction: the mobility peaks of the compounds of interest were first extracted from the regions of the mass spectra specific to each of the targeted compounds (extracted ion mobilograms (XIM)). The obtained XIM were then automatically integrated to retrieve the peak areas using MassLynx software [9,12], and the normalized responses were calculated using the ratio of SLX mobility peak area to its IS mobility peak area.

## 3. Results

### 3.1. Determination of the Mass of Parent and Fragment Ions of Selinexor (SLX) and [^2^H_5_]-SLX

The first MALDI-MS analysis of the parent and fragment ions of selinexor (SLX) and its labeled internal standard ([^2^H_5_]-SLX) was performed in order to identify potential contaminants from the MALDI matrix or from the compounds themselves that could interfere with the detection of SLX and [^2^H_5_]-SLX.

**Figure 2:**
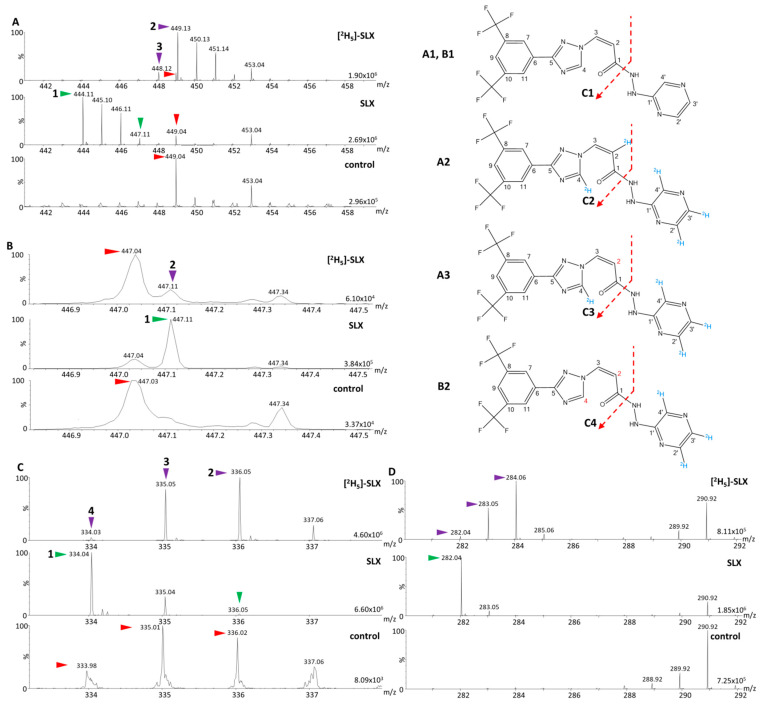
Characterization of selinexor (SLX) and the possible internal standard (IS), [^2^H_5_]-SLX, without biological matrix. (**A**) Mass spectrometry (MS) spectra without fragmentation (parent ions) of [^2^H_5_]-SLX and SLX in solution, and of the blank MALDI matrix solution (control). (**B**) Zoomed MS spectra of *m/z* 447 for the [^2^H_5_]-SLX and SLX solutions, and for the blank matrix solution. (**C**,**D**) MS spectra with fragmentation (MS/MS) of the [^2^H_5_]-SLX and SLX solutions showing the main fragments of SLX at (**C**) *m/z* 334.05 and (**D**) *m/z* 282.05 and the associated fragments of [^2^H_5_]-SLX, as well as the control MS/MS spectra of the blank matrix solution. Peaks of interest of the reference standard SLX are marked with green arrows, peaks of interest of the two candidates for IS are marked with purple arrows, and interfering peaks are marked with red arrows. (**A1**–**A3**) and (**B1**,**B2**) correspond to the structures of the parent compounds visible in the spectra from insets A and B, respectively. (**C1**–**C4**) correspond to fragments visible in the spectra from inset (**C**).

The analyses of the parent compound solutions (Figure 2A,B) indicated that the signal of the monoisotopic ion of the completely labeled [^2^H_5_]-SLX (*m/z* 449.13, Figure 2(A2)) did not overlap with any isotope of SLX, but interference from the MALDI matrix in its vicinity was detected (*m/z* 449.04). The incompletely labeled [^2^H_5_]-SLX at C2 was also detected at *m/z* 448.12 (Figure 2(A3)). A discrete signal at *m/z* 447.12 was observed, suggesting the presence of another partially unlabeled form of the IS (Figure 2(B2)). In MS/MS, the following major monoisotopic fragments were produced: *m/z* 334.04 (Figure 2(C1)) and *m/z* 282.04 for SLX, and *m/z* 335.05 (Figure 2(C3)), *m/z* 336.06 (Figure 2(C2)), and *m/z* 284.06 for [^2^H_5_]-SLX (Figure 2C,D). The peak from the [^2^H_5_]-SLX fragment at *m/z* 335.05 overlapped with the second isotopic peak of the major fragment of SLX (*m/z* 335.04), and the [^2^H_5_]-SLX MS/MS spectrum exhibited a peak at *m/z* 334.05 (Figure 2(C4)) that could represent a minor interference with the SLX monoisotopic signal (Figure 2C). The peak at *m/z* 334.05 obviously corresponded to the fragment of a partially unlabeled form of the IS at C2 and C4, originating from the related parent ion that was observed as a discrete peak at *m/z* 447.12 in the solution of IS (Figure 2(B2)).

**Figure 3:**
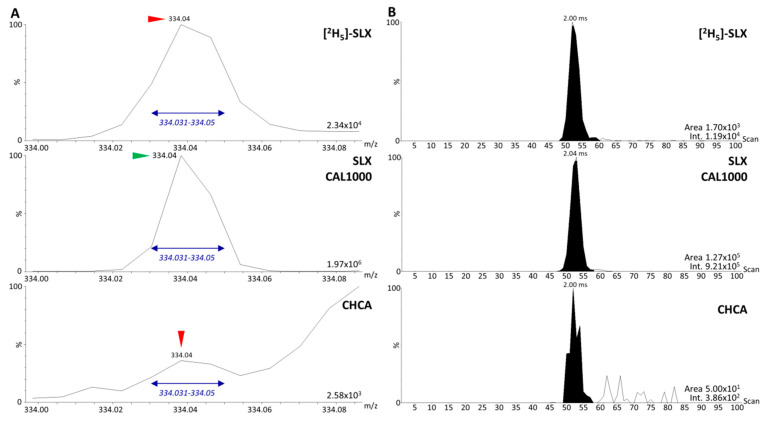
Estimation of the origin and level of interference caused by the *m/z* 334.05 peak in the [^2^H_5_]-selinexor (SLX) signal by comparison with the SLX and blank MALDI matrix (CHCA) signals. (**A**) The *m/z* range 334.00–334.10 of the MS/MS spectra of [^2^H_5_]-SLX, SLX, and CHCA. The *m/z* window used to retrieve the extracted ion mobilograms (XIM) is indicated in blue. (**B**) XIM of [^2^H_5_]-SLX, SLX, and CHCA with the results of the automatic peak integration and the associated signal intensities. Peaks of interest of the reference compound SLX are marked with green arrows and interfering peaks are marked with red arrows.

The second fragment detected for SLX (*m/z* 282.05) did not interfere with the related [^2^H_5_]-SLX monoisotopic fragment (Figure 2D), but the intensities of these second fragments were three to six times lower than the intensities obtained for the main fragments (*m/z* 334.05 for SLX and *m/z* 336.06 for [^2^H_5_]-SLX). Because our previous experience indicated that the most abundant fragments should be used to develop the most sensitive MS/MS assays for drug quantification using MALDI, we focused on the major fragment from [^2^H_5_]-SLX displaying the least interference with SLX, namely *m/z* 336.06. To ensure that the minor interfering signal observed on the MS/MS spectrum of [^2^H_5_]-SLX (*m/z* 334.05) would not hinder the reliable quantification of SLX, we used the MobA data extraction method, which is currently the most reliable method for quantitative DI assays using IM-MS [2]. We evaluated the areas of the XIM peaks from the monoisotopic peak of SLX and from the overlapping [^2^H_5_]-SLX signal (Figure 3A,B).

The comparison between CHCA alone and [^2^H_5_]-SLX revealed a 34-fold higher area for the interfering signal in [^2^H_5_]-SLX (Figure 3B), thus confirming that the interference originated from [^2^H_5_]-SLX itself and not from the MALDI matrix. This could be explained by an incomplete incorporation of ^2^H at C4, as expected from the certificate of analysis of this compound. Additionally, the area of the mobility peak of *m/z* 334.05 from [^2^H_5_]-SLX corresponded to 1% of the area of the mobility peak of *m/z* 334.05 from the reference compound SLX at 1000 ng/mL in solution. At such a high concentration the interfering signal was then so weak that it did not interfere with the actual estimation of the SLX signal, but this would need to be checked at lower concentrations to carefully choose the lower limit of reliable quantification (LLOQ).

### 3.2. Analysis of Dilution Series of Selinexor in Biological Matrix and Calibration Using Selinexor Fragments

**Figure 4:**
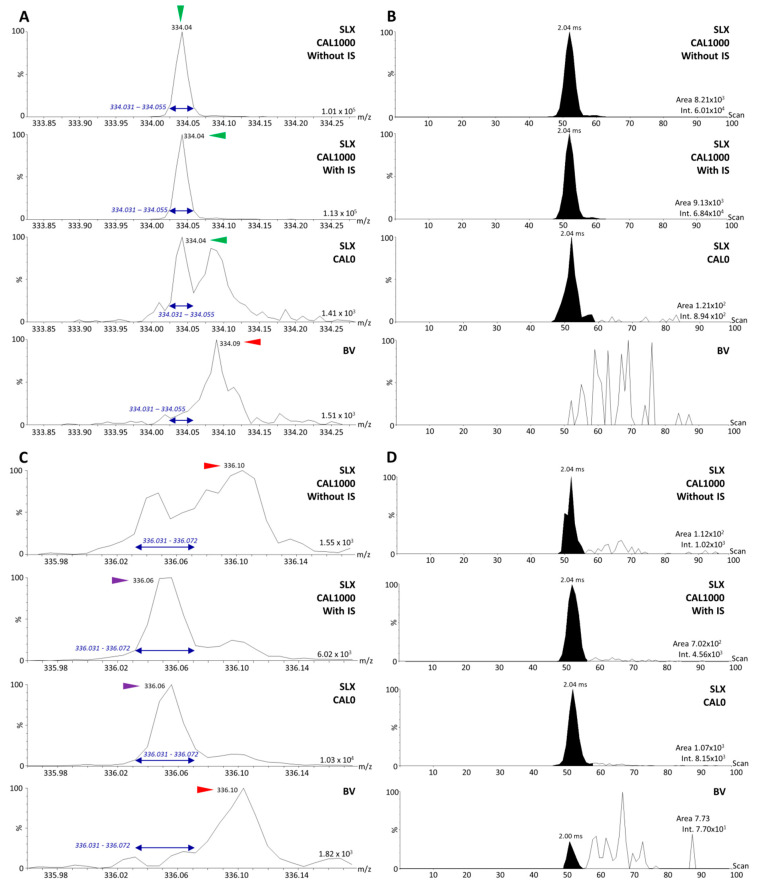
Estimation of the level of interference between fragments of selinexor (SLX) and of its internal standard (IS), [^2^H_5_]-SLX, in the biological matrix. (**A**) The *m/z* range 333.8-334.3 of the MS/MS spectra of a CAL1000-like sample (1000 ng/mL SLX in blank biological matrix without IS), a CAL1000 sample, a CAL0 sample (blank biological matrix spiked with IS only), and a blind value (BV) sample (blank biological matrix). The *m/z* window used to retrieve the extracted ion mobilograms (XIM) is indicated in blue. (**B**) XIM of the *m/z* 334.04 peak for CAL1000-like, CAL1000, CAL0, and BV samples with the results of the automatic peak integration and the associated signal intensities. (**C**) The *m/z* range 335.96-336.18 of the MS/MS spectra of the CAL1000-like, CAL1000, CAL0, and BV samples. The *m/z* window used to retrieve the XIM is indicated in blue. (**D**) XIM of the *m/z* 336.06 peak for CAL1000-like, CAL1000, CAL0, and BV samples with the results of the automatic peak integration and the associated signal intensities. Peaks of interest of the reference compound SLX are marked with green arrows, peaks of interest of the IS are marked with purple arrows, and interfering peaks are marked with red arrows.

As specified in pertinent regulatory guidelines [15,16,17], the signal of the targeted reference ion in the blind value (BV) and CAL0 samples (blank biological matrix and biological matrix spiked only with the IS, respectively) should be <20% of the lowest signal of the targeted ion in the LLOQ samples. The targeted IS signal in the BV should also not exceed 5% of the mean IS signal in the different CAL samples. However, no clear guidance is given regarding the maximum level of the interfering signal from the reference standard when measuring the IS signal. To minimize the influence of the interfering signal, a threshold similar to that of the BV (i.e., <5% of the mean IS signal from the CAL samples) was considered to be most appropriate. We aimed to evaluate the level of interference from the fragments of the IS, [^2^H_5_]-SLX, and of the reference standard, SLX, in a quantification context. Two possible sources of interference between the IS signal and the SLX signal remained to be verified: (i) the discrete overlapping signal from the IS with the monoisotopic ion of SLX at *m/z* 334.04 (Figure 4A,B) and (ii) the discrete overlapping signal from the third isotopic ion of SLX with the monoisotopic ion of the IS at *m/z* 336.06 (Figure 4C,D). In order to reduce the influence of the interference from the IS, we decreased its concentration by diluting it ten-fold before spiking the samples. The signal of the highest concentration point (CAL1000) was more than 75 times higher than in CAL0 (Figure 4A,B). An LLOQ above 65 ng/mL could then be expected when targeting the fragments at *m/z* 334.05 and *m/z* 336.06 for quantification. It was also important to estimate the level of interference from the signal of the third isotope of SLX in the highest calibration point when compared to the signal of the IS. Therefore, CAL0 was compared with a CAL1000-like sample without IS. The CAL0 sample displayed a 9.5 times more intense *m/z* 336.06 signal than the CAL1000-like sample containing no IS (Figure 4C,D). This needed to be further evaluated in order to see to what extent it might interfere with reliable quantification.

**Figure 5:**
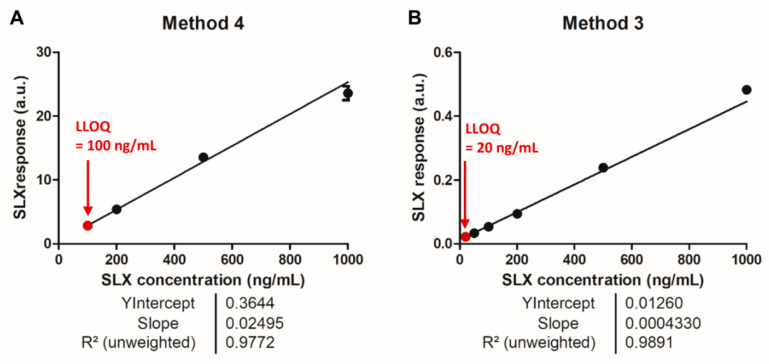
Calibration curves with 1/x^2^ weighing obtained from analyses of selinexor (SLX) calibration standard samples (**A**) using fragments of SLX and its internal standard, [^2^H_5_]-SLX (method 4), and (**B**) using the parent compounds (method 3). Lower limits of quantification (LLOQ) obtained for each method are indicated in red. Details of the parameters of the applied mass spectrometric methods are given in Table 2.

The calibration curve was built using method 4 (MS/MS) for the acquisition and the MobA data extraction method (retrieval of the areas of the mobility peaks specific to the targeted compounds from the XIM extracted from the targeted *m/z* windows (Figure 4A,C)). The LLOQ was derived on the basis of the following parameters: (i) the SLX peak area should be at least five times higher than the IS interfering signal in CAL0 and BV, (ii) the accuracy of the back-calculated concentrations should be within the recommended ±20% bias limits, and (iii) the precision between replicates should remain <20% CV (coefficient of variation). The analysis of the dilution series revealed an LLOQ of 100 ng/mL, as expected (Figure 5A).

The use of SLX and IS fragments at *m/z* 334-336 led to high interference and thus a high LLOQ and unreliable quantification at lower concentrations. The less intense fragments of SLX and its IS at *m/z* 282-284 had similar interference levels between isotopic peaks; therefore, using this minor fragment would not have helped to improve the LLOQ.

### 3.3. Analysis of the Dilution Series of Selinexor in Blank Matrix and Calibration Using the Parent Compounds

**Figure 6:**
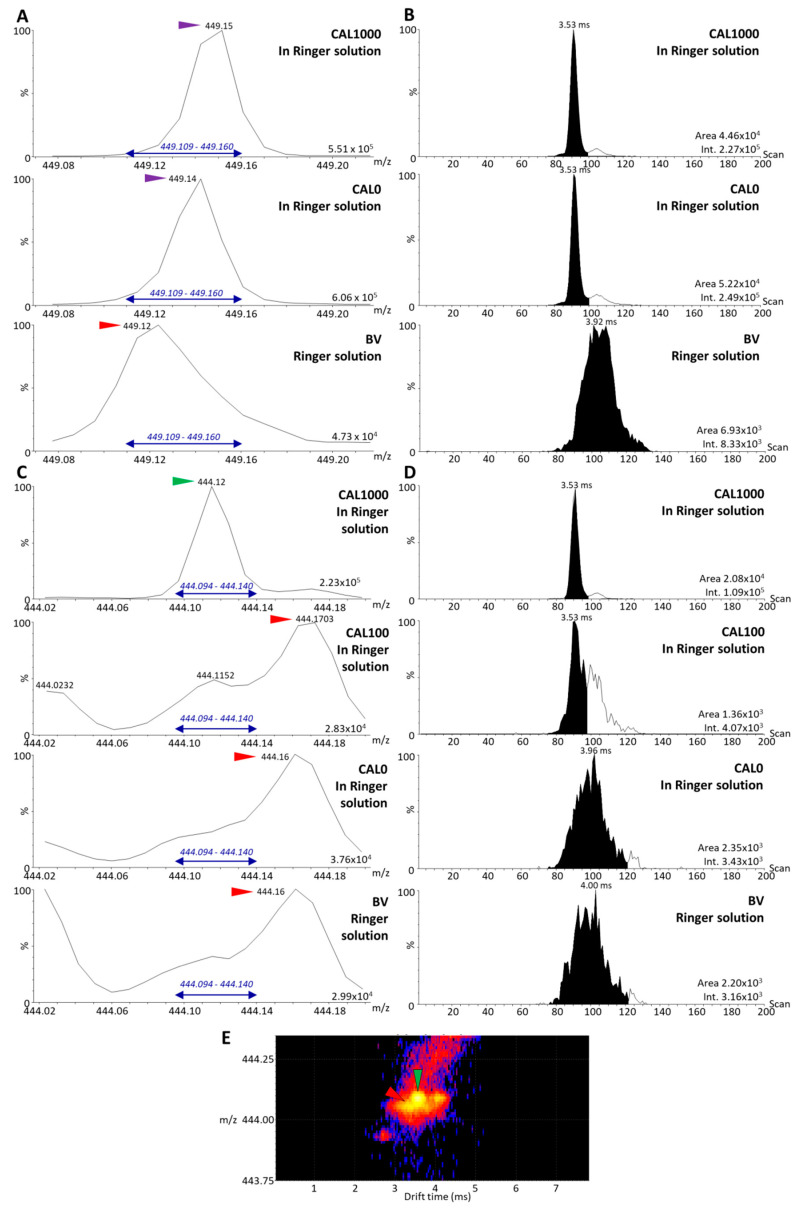
Estimation of the level of interference between parent selinexor (SLX) and parent internal standard (IS), [^2^H_5_]-SLX, in biological matrix. (**A**) The *m/z* range 449.00–449.25 of the MS spectra of a CAL1000 sample, a CAL0 sample (blank biological matrix spiked with IS only), and a blind value (BV) sample (blank biological matrix). The *m/z* window used to retrieve the extracted ion mobilograms (XIM) is indicated in blue. (**B**) XIM of the *m/z* 449.14 peak for CAL1000, CAL0, and BV samples with the results of the automatic peak integration and the associated signal intensities. (**C**) The *m/z* range 444.0–444.2 of the MS spectra of CAL1000, CAL100, CAL0, and BV samples. The *m/z* window used to retrieve the XIM is indicated in blue. (**D**) XIM of the *m/z* 444.12 peak for CAL1000, CAL100, CAL0, and BV samples with the results of the automatic peak integration and the associated signal intensities. (**E**) Two-dimensional mobility map of the SLX peak of a CAL1000 sample highlighting the presence of an interfering signal from the biological matrix. Peaks of interest of the reference compound SLX are marked with green arrows, peaks of interest of the IS are marked with purple arrows, and interfering peaks are marked with red arrows.

Although MALDI assays have been shown to be less sensitive when using parent compounds [9], using this approach in this particular context could reduce the impact of interference originating from both the IS and SLX, because the partially labeled compounds could be ignored in the analysis. CAL samples were prepared using the non-diluted working solution of the IS (500 ng/mL) and a new calibration curve was established using the IM-MS data (Figure 5B). In this case, the IS signal was high enough to be accurately integrated and distinguished from the background (Figure 6A,B). However, the area of the detected signal in the IS *m/z* window in the BV was >5% of the mean IS area. For further validation, this point would remain to be optimized. The analyses of the CAL and BV samples also indicated a very high background in the SLX *m/z* window (Figure 6C,D), which was confirmed by the 2D mobility map (Figure 6E), but no interference from the IS was observed. The analyses of the dilution series indicated that reliable calibration curves computed with a 1/x^2^ weighing could be obtained with acceptable linearity (R^2^ = 0.9891) from CAL20 (LLOQ 20 ng/mL, Figure 5B). In conclusion, although MALDI-MS-based assays for drug quantification were formerly found to be less sensitive than MS/MS-based assays [9], the former appears to be an alternative in the context of partial labeling of internal standards.

## 4. Discussion

The presented method development demonstrates the critical importance of the selection of the IS for the development of MALDI-MS and MS/MS assays relying on strategies that do not favor sensitive and accurate quantification using MRM or pseudo-MRM modes. MRM and related modes involve the monitoring of highly specific transitions between one parent ion and one derived fragment ion. As mentioned before, the use of such a quantification technique requires iterative analyses in micro-cycles within one acquisition to measure the different targeted compounds, and thus requires a homogeneous ion flow, as favored by ESI and liquid samples for instance. Using MALDI-MS, especially with MALDI matrices displaying heterogeneous crystallization, we have previously shown that the validation of MALDI-MS quantification methods could be challenging even when IS known to provide robust normalization were used [2]. MRM-like methods involve additional challenges of normalization. Because the signals of the IS are acquired independently of the drug signals, MRM data are thus more prone to artifacts due to MALDI matrix crystallization heterogeneity and consequent signal instability. MS/MS acquisition methods, such as the previously described method 4 [9], enable the detection of distinct signals of the ions of interest within the same analytical window. These may represent the most promising approaches for the development of more universal absolute MALDI-MS-based quantitative assays using internal standardization. When selecting the parent ions using the quadrupole, as performed in this application, it is crucial to know the resolution of the quadrupole to make sure that the IS ions will be selected together with the reference standard ions. The development of MS/MS methods (e.g., method 4) [9], and also MS methods using quadrupole selection of the parent ion (e.g., method 3) [9], necessitates careful selection of the IS depending on its MW, fragmentation pattern in the case of MS/MS, and labeling purity. In this article, we demonstrate that even a small proportion of unlabeled internal standard can dramatically increase the LLOQs of MS/MS-based methods, and consequently alter their orders of magnitude. One order of magnitude was indeed obtained in our context whereas previously up to three orders of magnitude could be obtained using MALDI-IM-MS/MS methods [9]. In this context, closely related compounds may be preferred as alternatives to isotopically labeled reference compounds (e.g., containing deuterium). In the specific context of SLX quantification, compounds such as eltanexor (MW = 428.29 g/mol) and verdinexor (MW = 442.3 g/mol) could be considered as IS candidates. However, the MW difference between eltanexor and SLX might be too large to select the signals of both compounds even with a low-resolution quadrupole. Conversely, verdinexor appears to have a MW that is too close to that of SLX (ΔMW = 1 g/mol), but using MS devices equipped with IM separation might help to clearly distinguish the SLX signal from the verdinexor signal despite the small ΔMW. Hence, the selection of a suitable IS to develop sensitive MALDI-MS assays for drug quantification must also consider the characteristics of the instrumentation. It is important to note that the selection of the IS belongs to the step of method development. Method application to drug development and clinical trials would necessitate further steps for full bioanalytical method validation according the US Food and Drug Administration (FDA) and the European Medicines Agency (EMA) guidelines. In this context, reproducibility of calibration linearity, inter- and intra-batch precision and accuracy, specificity, recovery, matrix effect, carry-over, and stability, among other relevant parameters, should be evaluated using a range of dedicated CAL and quality control (QC) samples [1].

In conclusion, the speed of MALDI-MS methods for drug quantification is offset by the challenges posed by the lack of chromatographic separation prior to ionization, and the diversity of the crystallization properties of MALDI matrices and their ionization efficiencies towards different compounds. In the present study, we demonstrated that the properties of the IS was another important parameter playing a major role in the sensitivity of the developed MALDI-MS assay.

## Figures and Tables

**Table 1:** Concentrations of selinexor (SLX) in the calibration standard (CAL) solutions prepared in solution and in biological matrix.

Calibration Point	Concentration in Solution (ng/mL)	Concentration in Biological Matrix (ng/mL)
CAL1000	2000	1000
CAL500	1000	500
CAL200	400	200
CAL100	200	100
CAL50	100	50
CAL20	40	20
CAL10	20	10
CAL5	10	5
CAL0	0	0
Blind value (BV)	0	0

**Table 2:** Summary of the acquisition methods tested for the quantification of SLX in microdialysis fluid using MALDI-IM-MS and MALDI-IM-MS/MS.

Method	Quadrupole	Collision Energy (eV)	Ion Mobility	Target Ion SLX (*m/z*)	Internal Standard	Target Ion IS (*m/z*)
3	*m/z* 444	0	✓	444.11	SLX-d_5_	449.13
4	*m/z* 444	32	✓	444.11	SLX-d_5_	449.13

## Data Availability

Not applicable.

## Sample Availability

Samples of the compounds are not available.
